# Loss of junctional plakoglobin (JUP) activates PI3K/AKT signaling in head and neck squamous cell carcinoma

**DOI:** 10.1007/s12032-026-03277-8

**Published:** 2026-02-19

**Authors:** Marius Hörner, Natalie Burkard, Babak Saravi, Timo Kohler, Andreas Vollmer, Matthias Kelm, Julian Volland, Tobias Renner, Alexander Kübler, Kai Kretzschmar, Nicolas Schlegel, Stefan Hartmann

**Affiliations:** 1 Department of Oral and Maxillofacial Plastic-Head and Neck Surgery, University Hospital of Würzburg, Pleicherwall 2, 97070 Würzburg, Germany; 2 Department of General, Visceral, Vascular and Pediatric Surgery, University Hospital Würzburg, Würzburg, Germany; 3 Department of Oral, Maxillofacial and Facial Plastic Surgery, Medical Faculty and University Hospital Düsseldorf, Heinrich-Heine-University Düsseldorf, 40225 Düsseldorf, Germany; 4 Department of Biochemistry, University of Cambridge, Tennis Court Road, Cambridge, CB2 1GA UK; 5 Institute of Anatomy and Experimental Morphology, University Medical Center Hamburg- Eppendorf, Martinistr. 52, 20246 Hamburg, Germany; 6 Mildred Scheel Early Career Centre (MSNZ) for Cancer Research Würzburg, University Hospital Würzburg, 97080 Würzburg, Germany

**Keywords:** HNSCC, Proliferation, PI3K/AKT signaling, Junctional plakoglobin

## Abstract

Head and neck squamous cell carcinomas (HNSCC) are characterized by poor prognosis, primarily due to early metastatic spread. The junctional plaque protein plakoglobin (JUP), a key desmosomal component, contributes not only to cell–cell adhesion but also to intracellular signaling processes that regulate proliferation, migration, and metastasis. Although JUP has been implicated in tumor progression in other cancer types, its role in HNSCC remains largely undefined. In this study, clinical data from The Cancer Genome Atlas (TCGA) were analyzed to determine the prognostic relevance of JUP expression in HNSCC. Functional studies were performed in HPV-negative (FaDu) and HPV-positive (UPCI-SCC-154) cells following siRNA-mediated JUP knockdown, assessing proliferation, wound healing, and signaling activity. Pharmacological inhibition experiments were conducted to evaluate pathway specificity. Low JUP expression was significantly associated with advanced metastatic stage and reduced overall survival in HNSCC patients. In vitro, JUP-deficient cells exhibited accelerated wound closure and increased proliferation. Mechanistically, loss of JUP led to activation of the phosphoinositide 3-kinase (PI3K)/AKT signaling pathway, and the enhanced motility phenotype could be reversed by pharmacological PI3K inhibition. Taken together, these findings identify plakoglobin as a negative regulator of PI3K signaling in HNSCC. Loss of JUP promotes tumor cell motility and proliferation, underscoring its potential value as a prognostic biomarker and therapeutic target in head and neck cancer.

**Supplementary Information:**

The online version contains supplementary material available at 10.1007/s12032-026-03277-8.

## Introduction

Head and neck squamous cell carcinoma (HNSCC) represents the sixth most common cancer worldwide, with nearly 900,000 new cases annually and mortality rates approaching 50% [1, 2]. The high lethality is primarily due to late-stage diagnosis, loco-regional recurrence, and resistance to current therapeutic regimens. Despite advances in surgery, radiotherapy, chemotherapy, and immunotherapy, treatment options remain limited, particularly for patients with recurrent or metastatic disease [3].

The incidence of oral cancer is closely associated with detrimental oral-related behaviors such as tobacco smoking and excessive alcohol consumption, as well as exposure to human papillomavirus (HPV) [1].

Despite therapeutic advances, prognosis remains poor, largely due to the high propensity for early lymphatic spread, local recurrence, and resistance to conventional therapies. While considerable progress has been made in elucidating key oncogenic drivers, the molecular mechanisms underlying the invasive phenotype of HNSCC remain only partially understood.

A potentially critical but underexplored regulator in this context is junctional plakoglobin (JUP), a junctional plaque protein and central component of the desmosomal complex [4]. Desmosomes are traditionally regarded as static mechanical adhesion units maintaining epithelial integrity. However, recent evidence indicates that desmosomes also function as dynamic signaling hubs, modulating diverse cellular processes including proliferation, migration, apoptosis, and epithelial morphogenesis [5, 6]. Altered expression of desmosomal proteins has been linked to cancer progression and poor clinical outcomes in various tumor entities [7–9]. Yet, the roles of individual desmosomal components in cancer appear to be context-dependent and, at times, paradoxical: acting either as tumor suppressors or as facilitators of malignancy [10–12]. JUP, in particular, has been implicated in epithelial carcinomas such as prostate and colorectal cancer, where it modulates oncogenic signaling cascades and contributes to metastatic spread [13, 14]. However, its precise role in HNSCC pathobiology has not been clarified.

In this study, we investigate the functional role of JUP in HNSCC progression. By employing siRNA-mediated knockdown models in HPV-negative and HPV-positive HNSCC cells, we examine how loss of JUP affects cellular proliferation and motility. Particular attention is given to the phosphoinositide 3-kinase (PI3K)/AKT pathway, a major oncogenic driver in HNSCC. Our findings aim to elucidate the contribution of desmosomal signaling to the invasive behavior of HNSCC and explore the potential of JUP as a prognostic marker and therapeutic target in epithelial malignancies.

## Materials and methods

### Antibodies and reagents

All antibodies and reagents w Tables 1 and 2.

**Table 1:** Primary and secondary antibodies with dilutions used for Western blotting (WB) and Immunofluorescence staining (IS)

Antibody	Source	Catalogue Number	WB	IS
Alexa Fluor 488	Thermo Fisher Scientific, Waltham, USA	A-21,201	n/a	1:200
Cy3	Dianova, Hamburg, Germany	115-165-003	n/a	1:600
Ki-67	Abcam, Cambridge, UK	ab15580	1:500	1:200
DAPI	Calbiochem, Darmstadt, Germany	268,298	n/a	1:1000
JUP	Cell signaling, Danvers, USA	2101	1:1000	n/a
Phospho AKT (SER473)	Cell Signaling, Leiden, Netherlands	4060 S	1:1000	n/a
β -actin	Abcam, Cambridge, UK	ab8226	1:1000	
p-FAK	Cell signaling, Danvers, USA	3284 S	1:1000	
FAK	Cell signaling, Danvers, USA	3285 S	1:1000	

**Table 2:** Test reagent used in this study are shown including the concentration applied in vivo and *in vitro*, respectively

Reagent	Source	Catalogue Number	Applied concentration *in vitro*
PI3K inhibitor (LY294002)	Millipore, Darmstadt, Germany	450 − 44	100ng/ml

### Cell culture

Human HNSCC cell lines were obtained from the American Type Culture Collection (ATCC). FaDu cells were cultured in MEM-α and F-12 medium supplemented with HEPES and 400 ng/mL hydrocortisone (Thermo Fisher Scientific, Waltham, MA, USA). SCC-154 were obtained from DSMZ (Braunschweig, Germany). UPCI-SCC-154 was cultured as described before: In MEM (with Earle’s salts) with additional 1% of non-essential amino acids [15]. Cell culture media were supplemented with 10% FCS and 1% Pen/Strep (Thermo Fisher Scientific, Waltham, MA, USA).

### siRNA-mediated silencing of JUP

FaDu cells were seeded in 6-well plates and cultured for 24 h prior to transfection, as previously described [16]. Human JUP-specific siRNA (Thermo Fisher Scientific, USA) was transfected using Lipofectamine LTX Reagent (Thermo Fisher Scientific, USA) according to the manufacturer’s protocol. A non-targeting siRNA (Abcam, UK) served as negative control. Transfection was initiated at 80% confluence. Based on preliminary experiments, effective JUP knockdown was observed at 48 h post-transfection, and all subsequent experiments were performed at this time point.

### Quantitative real-time PCR (qRT-PCR)

Total RNA was extracted from FaDu cells, and cDNA was synthesized using the iScript™ cDNA Synthesis Kit (Bio-Rad, Munich, Germany). Quantitative PCR was carried out with the MESA GREEN qPCR MasterMix Plus for SYBR^®^ Assay No ROX (Eurogentec, Cologne, Germany) on a CFX96 Touch Real-Time PCR Detection System (Bio-Rad, Munich, Germany). β-actin was used as the reference gene. All reactions were run in duplicate with an annealing temperature of 60 °C. Primer concentration was 5 µM; sequences are listed in Table 3.

**Table 3:** Primers used for quantitative real-time PCR

Primer	Species	Source	Primer sequences 5’-3’
*MKI67* forward	human	eurofins	CCT GTG AGG CTG AGA CAT GG
*MKI67* reverse	human	eurofins	CCC TCA CTC TTG TCA GGG TC
*AXIN2* forward	human	eurofins	CCA CAC CCT TCT CCA ATC C
*AXIN2* reverse	human	eurofins	TGC CAG TTT CTT TGG CTC TT

### Western blot

Cells were lysed and homogenized. Protein concentration was determined using a BCA protein assay kit (Thermo Fisher Scientific, USA), as previously reported [17]. Equal amounts of protein were separated by SDS-PAGE and transferred to nitrocellulose membranes. Membranes were incubated overnight at 4 °C with primary antibodies (see Table 1), followed by 1-hour incubation with species-specific secondary antibodies at room temperature. Protein signals were visualized using the SuperSignal West Pico PLUS Chemiluminescent Substrate (Thermo Fisher Scientific, USA). Densitometry was performed with ImageLab (Bio-Rad, Hercules, CA, USA) and normalized to β-actin.

### Immunofluorescence

Cell monolayers were fixed and incubated overnight at 4 °C with primary antibodies (see Table 1). Cy3- or Alexa Fluor 488-conjugated secondary antibodies (Dianova, Hamburg, Germany) were applied. Representative images were acquired using a confocal laser scanning microscope (Leica LSM 780, Zeiss, Oberkochen, Germany).

### In vitro wound healing assay

Cells were cultured in 6- or 8-well plates (Ibidi, Gräfelfing, Germany) until confluence. A linear scratch was introduced using a sterile 10 µL pipette tip by a blinded investigator, as previously described [18, 19]. After medium replacement, wound areas were imaged at 0-,24-, and 48-hours post-scratch. Wound closure was quantified using ImageJ software (Rasband, W.S., ImageJ, Bethesda, Maryland, USA) by measuring the residual scratch area relative to baseline (t = 0).

### TCGA analysis

Clinical and RNA-seq data for HNSCC were retrieved from The Cancer Genome Atlas (TCGA-HNSCC project). Transcript expression data (TPM normalized) were transformed to log2(TPM + 1) values for variance stabilization. Only “Primary Solid Tumor” and “Solid Tissue Normal” samples were included, yielding a dataset of 564 samples. Expression levels of JUP were analyzed. Shapiro-Wilk tests assessed normality in expression distributions. Parametric (unpaired t-test) or non-parametric (Mann–Whitney U test) tests were applied accordingly. Expression differences were visualized via violin plots with overlaid boxplots, and statistical significance was annotated.

### Statistical analysis

Statistical analysis was performed using GraphPad Prism (GraphPad Software, La Jolla, CA, USA). Data are expressed as means ± SEM. Statistical significance was set at *p* < 0.05. For two-group comparisons, paired Student’s t-tests or Mann-Whitney U tests were used, based on distribution normality (Shapiro-Wilk test). For multiple group comparisons, one-way ANOVA followed by Tukey’s or Šidák’s multiple comparisons test was used, or Kruskal-Wallis test for non-Gaussian data. The statistical test used in each case is indicated in the corresponding figure legend.

## Results

### Reduced *JUP* expression correlates with advanced nodal disease and poor survival

To assess the clinical relevance of JUP expression in head and neck squamous cell carcinoma (HNSCC), we analyzed RNA-sequencing data from 564 tumor and normal tissue samples in the TCGA-HNSCC cohort.

**Fig. 1:**
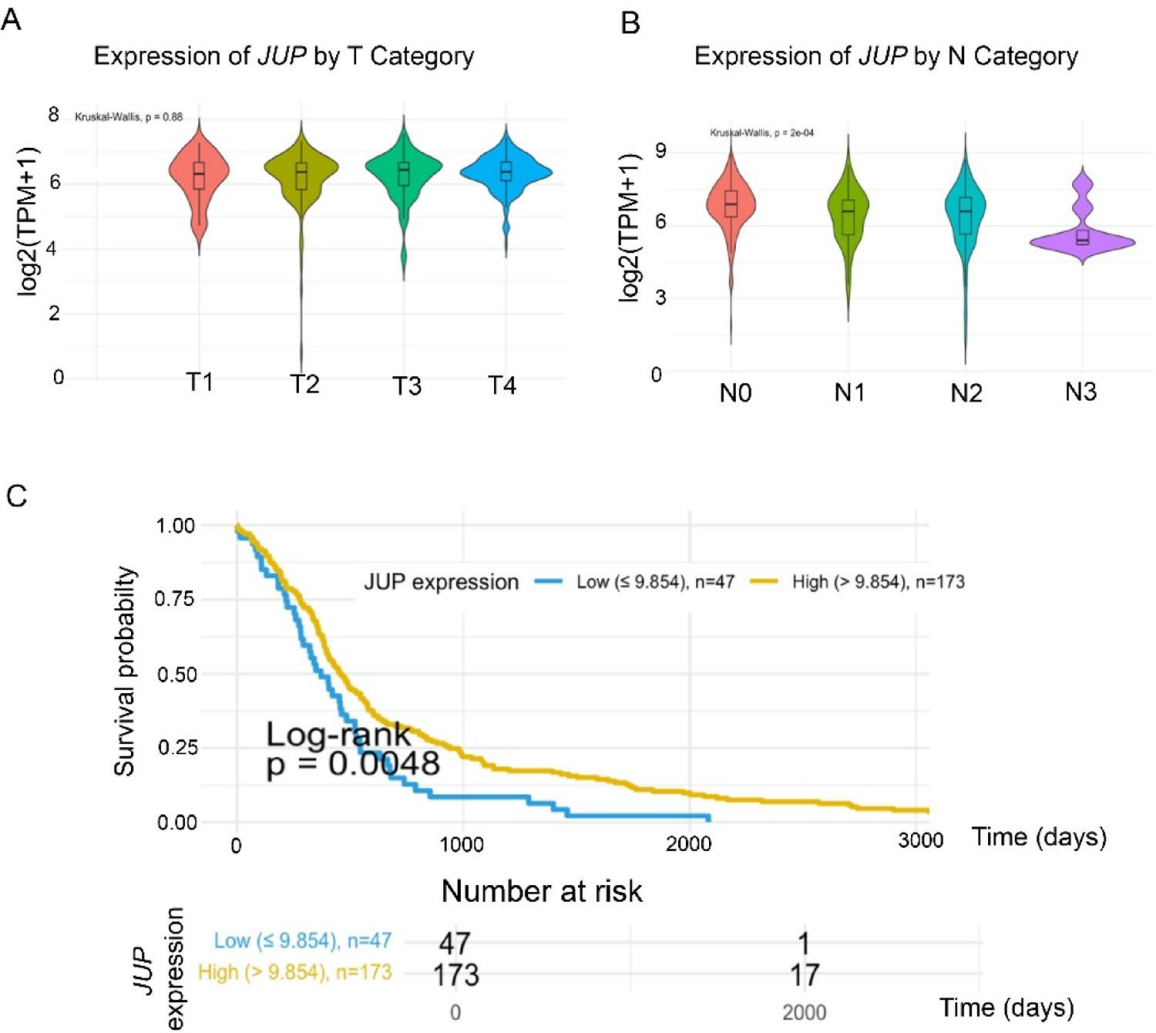
**A**) Expression of JUP (log₂[TPM + 1]) across different tumor T stages (T1, T2 ,T3 and t4) in the TCGA-HNSCC cohort. No statistically significant differences were observed among T categories (Kruskal–Wallis test, p = 0.88). **B**) Violin and boxplot overlay for JUP expression (log2(TPM+1)) among N0, N1, N2, and N3 primary tumors. A highly significant difference was observed (p = 0.00020) via Kruskal–Wallis, with N0 samples displaying the highest mean expression. Horizontal lines indicate median values, and boxplots reflect quartiles. **C**) Kaplan–Meier overall survival curves stratified by JUP expression. Patients with JUP ≤ 9.854 (log₂[TPM + 1]) were classified as low expression (blue), and those > 9.854 as high expression (gold). The log rank test p = 0.0048 indicates a significant difference in survival between the two groups. A table of numbers at risk is shown below the plot.

While no statistically significant differences in JUP expression were observed across tumor T stages (Fig. 1A; *p* = 0.88). JUP expression (log₂[TPM + 1]) was significantly associated with lymph node involvement (*p* = 0.0002; Fig. 1B). Mean JUP expression was highest in N0 tumors (10.522 ± 0.693) and progressively decreased in more advanced nodal stages, with the lowest levels detected in N3 tumors (5.816 ± 1.143). These data suggest that JUP expression is inversely correlated with nodal metastasis and may be involved in the regulation of lymphatic dissemination.

We next assessed whether *JUP* expression levels are associated with overall survival (OS). Kaplan-Meier analysis was performed for 220 primary HNSCC tumors with available survival data. Optimal cut-off values were determined using the maximally selected rank-statistics method, controlling for multiple testing. The identified *JUP* expression threshold was 9.854 (log₂[TPM + 1]); patients with lower expression exhibited significantly reduced OS (*p* = 0.0048; Fig. 1C). These results support a prognostic role for *JUP* in HNSCC.

### *JUP* knockdown enhances epithelial wound closure

**Fig. 2:**
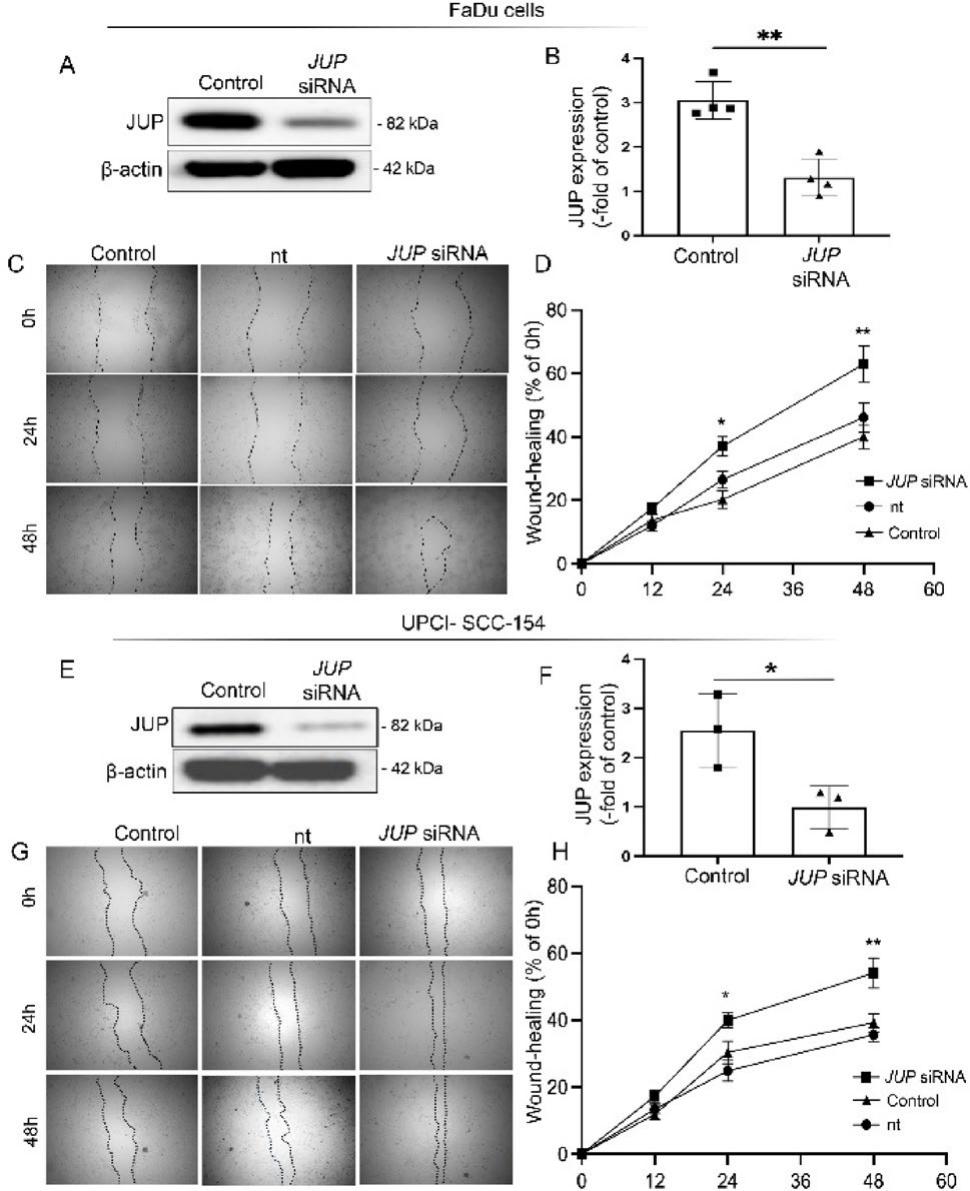
**A, B**) Representative Western blot is shown to verify the absence of JUP in FaDu cells after siRNA treatment. Membranes were reprobed for β-actin to verify equal protein loading (n=4). Data are means ± SEM Significance is determined by 1-way ANOVA followed by Tukey’s post hoc testing. *p≤ 0.05. **C**) Representative images from the FaDu wound healing assays at baseline (immediately after wounding), and at 24 hours and 48 hours post-wounding, are presented (n = 4), Scale bar is 250 µm. **D**) Quantification of the wound healing assays FaDu monolayers after 24 and 48 hours in relation to the initial wound area under the different conditions (control, non-target (nt), JUP siRNA) is shown, Data are shown as mean ± SEM (n = 4 experiments) and were analyzed by 1-way ANOVA followed by Tukey’s post hoc te sting. *p≤ 0.05. **E, F**) Representative Western blot is shown to verify the absence of JUP in SCC-154 cells after siRNA treatment. Membranes were reprobed for β-actin to verify equal protein loading (n=3) Data are means ± SEM Significance is determined by 1-way ANOVA followed by Tukey’s post hoc testing. *p≤ 0.05. **G**) Representative images from the FaDu wound healing assays at baseline (immediately after wounding), and at 24 hours and 48 hours post-wounding, are presented (n = 4), Scale bar is 250 µm. **H**) Quantification of the wound healing assays in SCC-154 monolayers after 24 and 48 hours in relation to the initial wound area under the different conditions (control, non-target (nt), JUP siRNA) is shown, Data are shown as mean ± SEM (n = 4 experiments) and were analyzed by 1-way ANOVA followed by Tukey’s post hoc testing. *p≤ 0.05.

To explore the functional significance of JUP in tumor cell behavior, we performed siRNA-mediated knockdown in FaDu cells. Efficient silencing of JUP was confirmed by Western blot analysis (Fig. 2A, B; control: 3.05 ± 0.42% vs. JUP siRNA: 1.31 ± 0.42%, *p* < 0.01). In scratch wound assays, JUP-depleted FaDu cells exhibited a markedly accelerated wound closure after 24 h and 48 h compared with both control and non-targeting (nt) control cells (Fig. 2C, D). After 24 h, wound closure reached 44.9 ± 7.3% in JUP-knockdown cells, compared with 24.6 ± 6.8% in controls and 27.8 ± 5.7% in nt controls (*p* < 0.05). At 48 h, wound closure was 45.1 ± 6.2% in JUP-siRNA cells, compared with 32.8 ± 7.6% in controls and 33.0 ± 5.9% in nt controls (*p* < 0.01), confirming a sustained motility-enhancing effect following JUP depletion.

To further validate these findings in an additional cell model, we performed complementary experiments in HPV-positive UPCI-SCC154 cells. After establishing an efficient JUP knockdown using siRNA (Fig. 2E, F), we next conducted scratch wound assays to assess the effect of JUP depletion on cell motility. Consistent with our previous results in FaDu cells, JUP-silenced SCC-154 cells exhibited markedly accelerated wound closure. After 24 h, wound closure was 19.7 ± 4.6% in JUP-siRNA cells, 12.5 ± 5.2% in nt controls, and 13.8 ± 4.3% in controls (*p* < 0.05). After 48 h, wound closure reached 44.9 ± 7.3% in JUP-knockdown cells, compared with 24.6 ± 6.8% in controls and 27.8 ± 5.7% in nt controls (*p* < 0.01). Collectively, our findings demonstrate that JUP depletion induces consistent phenotypic alterations across both HPV-negative (FaDu) and HPV-positive (SCC154) HNSCC cell models, suggesting a broader functional role of JUP in regulating tumor cell properties independent of HPV status.

### Increased Ki-67 expression indicates enhanced proliferation following *JUP* knockdown

To determine whether the increased wound closure observed upon JUP loss was associated with enhanced cell proliferation, we examined Ki-67 expression as a proliferation marker.

**Fig. 3:**
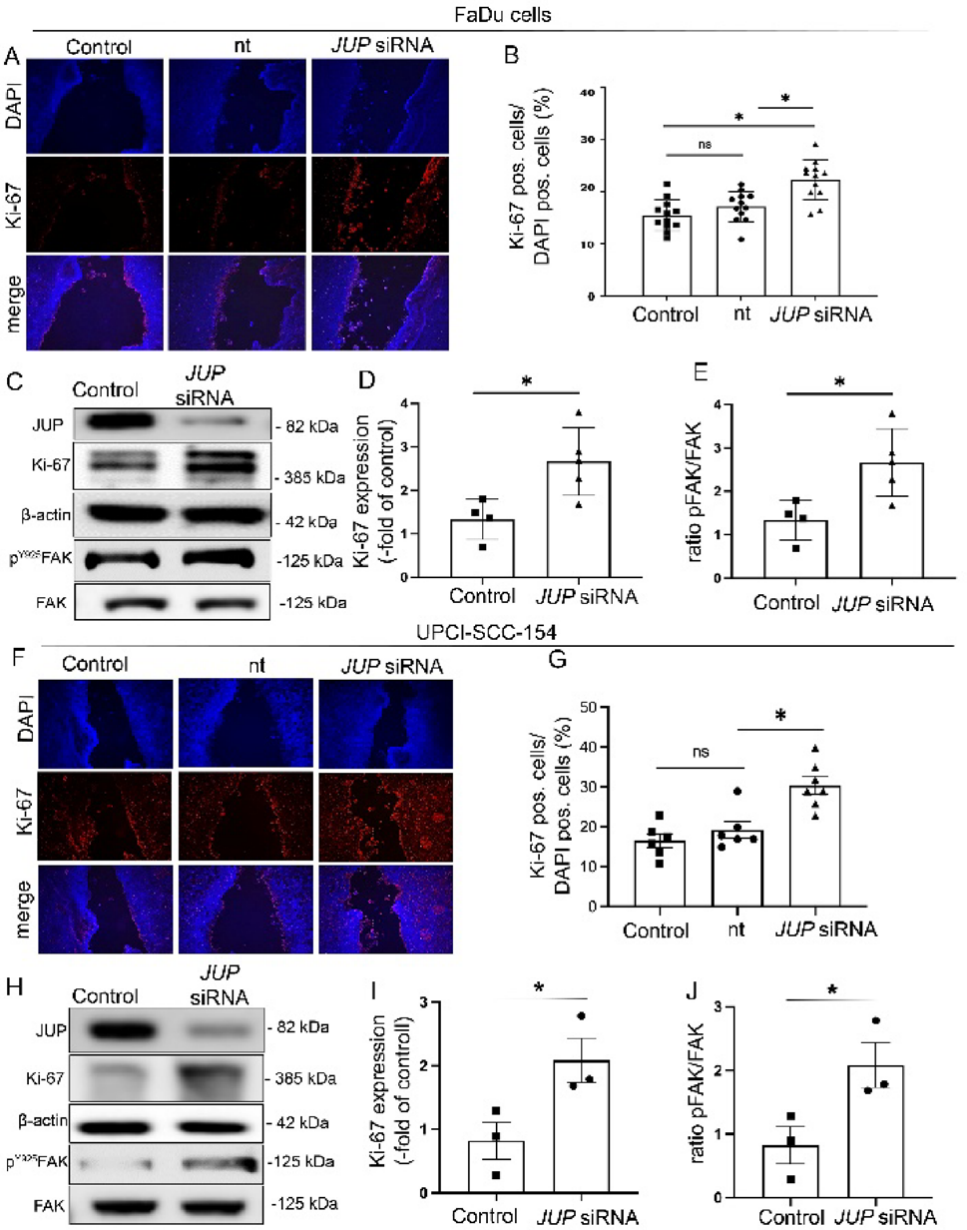
3 **A**) Quantification of proliferating cells (Ki67-positive/DAPI-positive) is presented as a ratio determined in scratch assays. **B**) Each dot represents the mean value from one FaDu scratch (n = 4). Data are shown as mean ± SEM. Statistical significance was assessed using a two-tailed Student’s t test; *p ≤ 0.05 was considered significant. **C**) Representative Western blots from FaDu cells 24h after scratch for Ki67 is shown. Membranes were reprobed with β-actin as loading control (n = 4). **D**) Western blot quantifications present data as relative protein levels compared to β-actin. Quantification confirms increased levels of Ki67 after loss of JUP. Data are shown as mean ± SEM (n = 4 experiments) and were analyzed by 1-way ANOVA followed by Tukey’s post hoc testing. **p≤ 0.005. **E**) Western blot quantifications are presented as relative protein levels normalized to total FAK. Data are shown as mean ± SEM (n = 4 independent experiments) and were analyzed using one-way ANOVA followed by Tukey’s post hoc test (p ≤ 0.005). **F**) Quantification of proliferating cells (Ki67-positive/DAPI-positive) is presented as a ratio determined in scratch assays. **G**) Each dot represents the mean value from one SCC-154 scratch (n = 4). Data are shown as mean ± SEM. Statistical significance was assessed using a two-tailed Student’s t test; *p ≤ 0.05 was considered significant. **H**) Representative Western blots from SCC-154 cells 24h after scratch for Ki67 is shown. Membranes were reprobed with β-actin as loading control (n = 4). **I**) Western blot quantifications present data as relative protein levels compared to β-actin. Quantification confirms increased levels of Ki67 after loss of JUP. Data are shown as mean ± SEM (n = 4 experiments) and were analyzed by 1-way ANOVA followed by Tukey’s post hoc testing. **p≤ 0.005.

Immunostaining 24 h after scratch, revealed a significantly higher percentage of Ki-67–positive cells in JUP-silenced monolayers (2.67 ± 0.78%) compared with controls (1.33 ± 0.46%; *p* < 0.01; Fig. 3A, B). This finding was further corroborated by Western blot and qPCR analyses, which confirmed increased Ki-67 expression at both the protein- (Fig. 3C, D) and mRNA levels (**Suppl. Figure A**) following JUP knockdown (mRNA: control = 1.22 ± 1.56 vs. *JUP* siRNA = 2.87 ± 1.31; *p* < 0.01). It should be noted that epithelial regeneration and tumor progression generally involve a combination of cell proliferation and motility-related processes [20]. To further dissect the potential contribution of cell migration, we analyzed phosphorylation of focal adhesion kinase (FAK) as a surrogate marker of migratory activity.

Western blot analyses demonstrated significantly elevated FAK phosphorylation in JUP-depleted cells relative to controls values (Fig. 3E).

Collectively, these results indicate that loss of JUP activates pathways linked to both proliferation and migration.

To further explore the mechanisms underlying the accelerated wound closure observed in HPV-positive SCC154 cells, we examined potential changes in cell proliferation.

Immunostaining revealed a significant increase in Ki-67-positive cells following JUP depletion (31.04 ± 4.68%) compared with control (18.78 ± 4.16%) and non-targeting (nt) controls (16.83 ± 3.13%) (*p* < 0.01; Fig. 3F, G). This observation was further confirmed at the protein level by Western blot analysis, which revealed elevated KI-67 expression, followed by increased FAK phosphorylation after JUP depletion (2.14 ± 3.89) compared with control cells (0.87 ± 3.72; Fig. 3H-J).

### Loss of JUP activates PI3K/AKT signaling in HPV-negative and HPV-positive HNSCC cell models

To further investigate the molecular mechanisms by which JUP regulates cellular proliferation and migration, we examined activation of the PI3K/AKT signaling pathway.

**Fig. 4:**
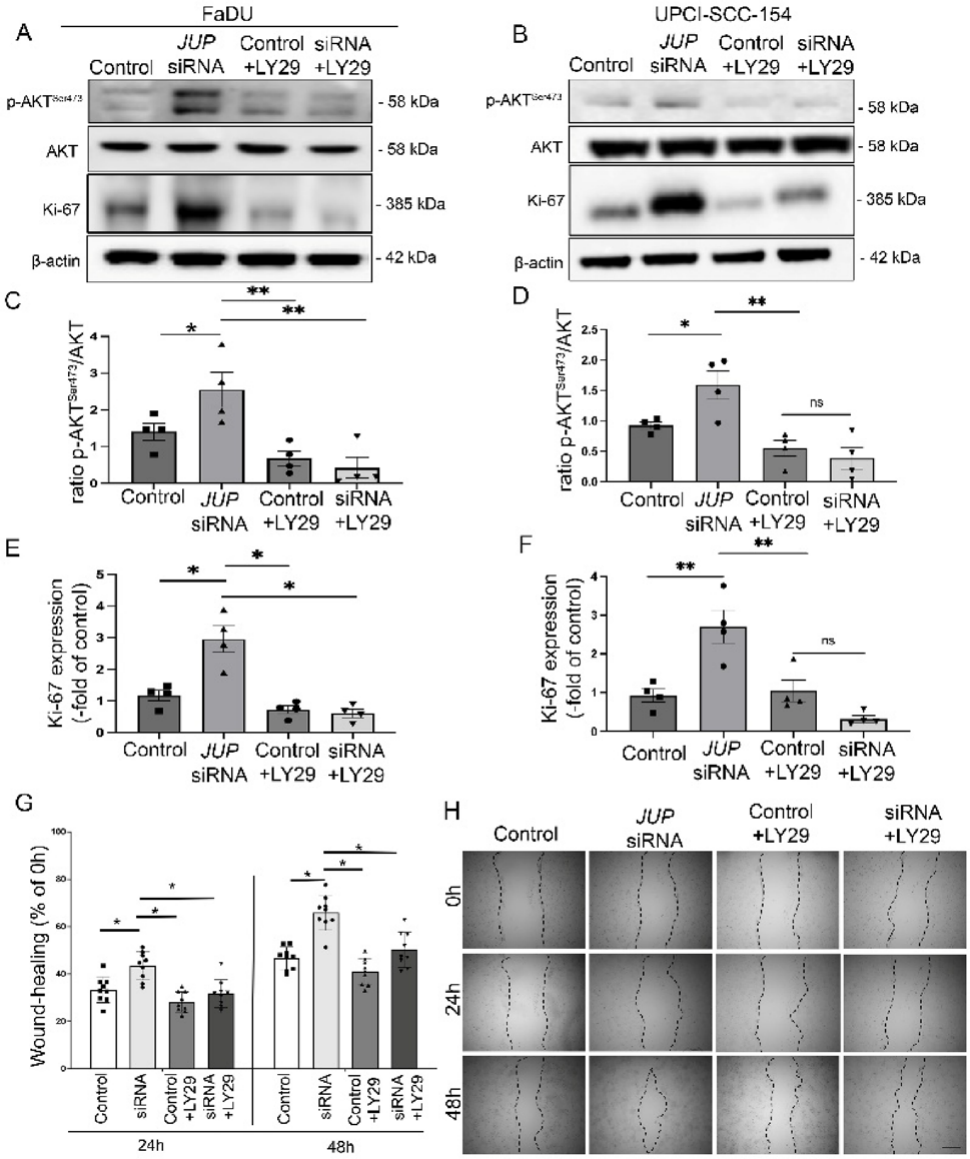
**A**) Representative Western blot showing phospho-AKT (Ser473), total AKT, Ki-67 and β-actin levels in control and JUP siRNA-treated FaDu cells, with or without LY294002 (LY29) treatment. **B**) Representative Western blot showing phospho-AKT (Ser473), total AKT, Ki-67 and β-actin levels in control and JUP siRNA-treated SCC-154 cells, with or without LY294002 (LY29) treatment. **C, D**) Quantification and statistical analysis of phospho-AKT levels (normalized to total AKT) and Ki-67 protein levels were performed in FaDu cells. Data are presented as mean ± SEM (n = 4) and were analyzed using one-way ANOVA followed by Tukey’s post hoc test. *p ≤ 0.05, **p ≤ 0.005. **E, F**) Quantification and statistical analysis of phospho-AKT levels (normalized to total AKT) and Ki-67 protein levels were performed in SCC cells. Data are presented as mean ± SEM (n = 4) and were analyzed using one-way ANOVA followed by Tukey’s post hoc test. *p ≤ 0.05, **p ≤ 0.005. **G**) Representative images of FaDu wound healing assays at 0 h, 24 h, and 48 h after scratch in the indicated treatment groups. Dotted lines indicate wound edges, Scale bar is 250 µm. **H**) Quantification of wound closure is shown below. Data are presented as mean ± SEM independent experiments (n = 4) and were analyzed using one-way ANOVA followed by Tukey’s post hoc test. *p ≤ 0.05 was considered significant.

Western blot analysis demonstrated that JUP knockdown resulted in increased levels of phosphorylated AKT (p-AKT) and the proliferation marker Ki-67 in both FaDu and SCC-154 cells (Fig. 4A, B).

Quantitative analysis revealed that p-AKT levels increased from 1.40 ± 0.49 in control cells to 2.55 ± 0.94 in JUP-siRNA–treated FaDu cells (Fig. 4C; *p* < 0.001) and from 1.23 ± 0.74 to 2.31 ± 1.12 in SCC-154 cells (Fig. 4D; *p* < 0.001).

Similarly, Ki-67 expression was elevated following JUP depletion, increasing from 1.17 ± 0.34 to 2.95 ± 0.83 in FaDu cells (Fig. 4E) and from 0.98 ± 0.57 to 2.68 ± 1.24 in SCC-154 cells (Fig. 4F).

To determine whether these effects were mediated by PI3K/AKT signaling, JUP-depleted cells were treated with the PI3K inhibitor LY294002. In both cell lines, PI3K inhibition markedly reduced p-AKT and Ki-67 levels, restoring them to near-control values (Fig. 4A–F), indicating PI3K/AKT-dependent regulation.

To assess whether these molecular changes translated into functional consequences, scratch wound assays were performed in FaDu cells, which displayed the strongest JUP-dependent activation of PI3K/AKT signaling.

JUP knockdown significantly accelerated wound closure compared with control conditions (Fig. 4G, H). Importantly, treatment with PI3K inhibitor (LY294002) completely abrogated this effect, restoring wound healing rates to control levels.

Together, these findings demonstrate that loss of JUP enhances proliferative and migratory behavior via activation of the PI3K/AKT pathway.

## Discussion

### *JUP* expression correlates with poor prognosis in HNSCC

Clinically, our analysis of TCGA data revealed that reduced *JUP* expression is significantly associated with advanced nodal disease and poor overall survival in HNSCC patients. Clinically, our TCGA analysis demonstrated that reduced JUP expression is significantly associated with advanced nodal disease and poor overall survival in HNSCC patients. This observation suggests that the relationship between JUP and the N stage may reflect a mechanistic role of JUP in metastatic dissemination rather than in primary tumor growth. Interestingly, recent data from the KEYNOTE-689 trial support this notion, showing that distant metastasis, rather than local tumor progression is the major determinant of poor prognosis in HNSCC [21]. In this context, the association of JUP downregulation with nodal disease may indicate an active role of JUP in metastatic spread.

These observations position JUP as a potential prognostic biomarker. Importantly, this aligns with findings from other epithelial cancers: for instance, in colorectal cancer, JUP-deficient cell clusters have been linked to organ-specific metastasis [14] and DSG2 overexpression has been associated with unfavorable outcomes in multiple tumor entities [22]. Our data thus suggest that plakoglobin may play a dual role: maintaining epithelial cohesion under physiological conditions while suppressing PI3K-driven proliferation and migration during malignant transformation. Loss of JUP may therefore create a permissive microenvironment for invasion and metastasis, which could explain its association with advanced clinical stages in HNSCC [14].

### Plakoglobin as a modulator of cellular signaling and phenotype in HNSCC

This study identifies plakoglobin (JUP), a key structural component of desmosomal junctions, as a functional regulator of epithelial cell proliferation and motility in head and neck squamous cell carcinoma (HNSCC). Using siRNA-mediated knockdown models, we demonstrate that loss of JUP enhances epithelial cell motility and proliferation through activation of the PI3K/AKT signaling pathway. These findings extend the biological role of plakoglobin beyond mechanical cohesion, uncovering a previously unrecognized function in oncogenic signaling and tumor progression.

### Loss of JUP promssotes epithelial proliferation and wound closure

Consistent with its structural role, JUP depletion in FaDu cells resulted in significantly accelerated wound closure and increased proliferation, as shown by scratch assays, Ki-67 immunostaining, and mRNA expression analysis. These results align with previous studies reporting that desmosomal protein loss impairs epithelial integrity and promotes proliferative repair responses [6, 23]. Similar findings have been described in various epithelial cancers, including those of the prostate and breast, where downregulation of JUP correlates with increased motility and invasive potential [24, 25]. Our data add to this growing body of evidence by highlighting the motility-promoting effects of JUP loss specifically in the HNSCC context.

### PI3K/AKT signaling as a mechanistic link

Mechanistically, we show that JUP depletion results in robust activation of the PI3K/AKT pathway, a signaling axis commonly altered in HNSCC and known to regulate epithelial proliferation and migration [26, 27]. PI3K inhibition with LY294002 reversed the motility and phosphorylation phenotype, confirming the functional relevance of this pathway. To our knowledge, this is the first demonstration of direct regulatory crosstalk between plakoglobin and the PI3K/AKT axis in head and neck cancer. These findings suggest that desmosomal proteins such as JUP may function as upstream modulators of kinase signaling and may thereby influence both tissue repair and tumorigenesis. Analogous mechanisms have been observed in other epithelial systems, such as the intestine, where loss of desmoglein-2 promotes aberrant kinase activation and disrupts tight junction dynamics [28, 29]. Our study supports the emerging view that desmosomal components actively participate in intracellular signaling and that their loss may trigger oncogenic reprogramming beyond structural destabilization [30].

### Therapeutic implications and future directions

These findings offer promising avenues for therapeutic intervention. The observation that PI3K inhibition mitigates the pro-migratory and proliferative phenotype of JUP-deficient cells suggests that PI3K pathway blockade may be beneficial in patients with reduced JUP expression. Moreover, the context-dependent role of JUP, acting both as a structural anchor at intercellular junctions and a signaling modulator, may inform personalized treatment strategies targeting tumor architecture and intracellular signaling simultaneously.

However, an important limitation of this study is the lack of in vivo validation. The results are currently restricted to cell culture experiments and TCGA data analysis. Future experiments using mouse xenograft or orthotopic HNSCC models are essential to definitively determine whether JUP loss promotes tumor progression and metastasis in vivo.

Further investigations should also aim to validate these findings in clinical specimens and to explore the upstream regulatory mechanisms governing JUP expression in epithelial tissues. In addition, the impact of JUP loss on immune modulation and therapeutic resistance in HNSCC remains to be elucidated.

## Conclusion

In summary, our study identifies plakoglobin (JUP) as a novel suppressor of PI3K/AKT signaling and epithelial cell motility in HNSCC. By bridging intercellular adhesion and intracellular signaling, JUP emerges as a key regulator of tumor progression. These insights expand the functional repertoire of desmosomal proteins and establish JUP as a potential prognostic marker and therapeutic target in head and neck cancer.

## Supplementary Information

Below is the link to the electronic supplementary material.

Supplementary Material 1

## Data Availability

The data that support the findings of this study are available from the corresponding author, M.H., upon reasonable request.
