# Supplementary figures and images for: Loss of junctional plakoglobin (JUP) activates PI3K/AKT signaling in head and neck squamous cell carcinoma
Source: Med Oncol. 2026 Feb 19;43(3):156. doi: 10.1007/s12032-026-03277-8 (PMC12920303; doi:10.1007/s12032-026-03277-8)

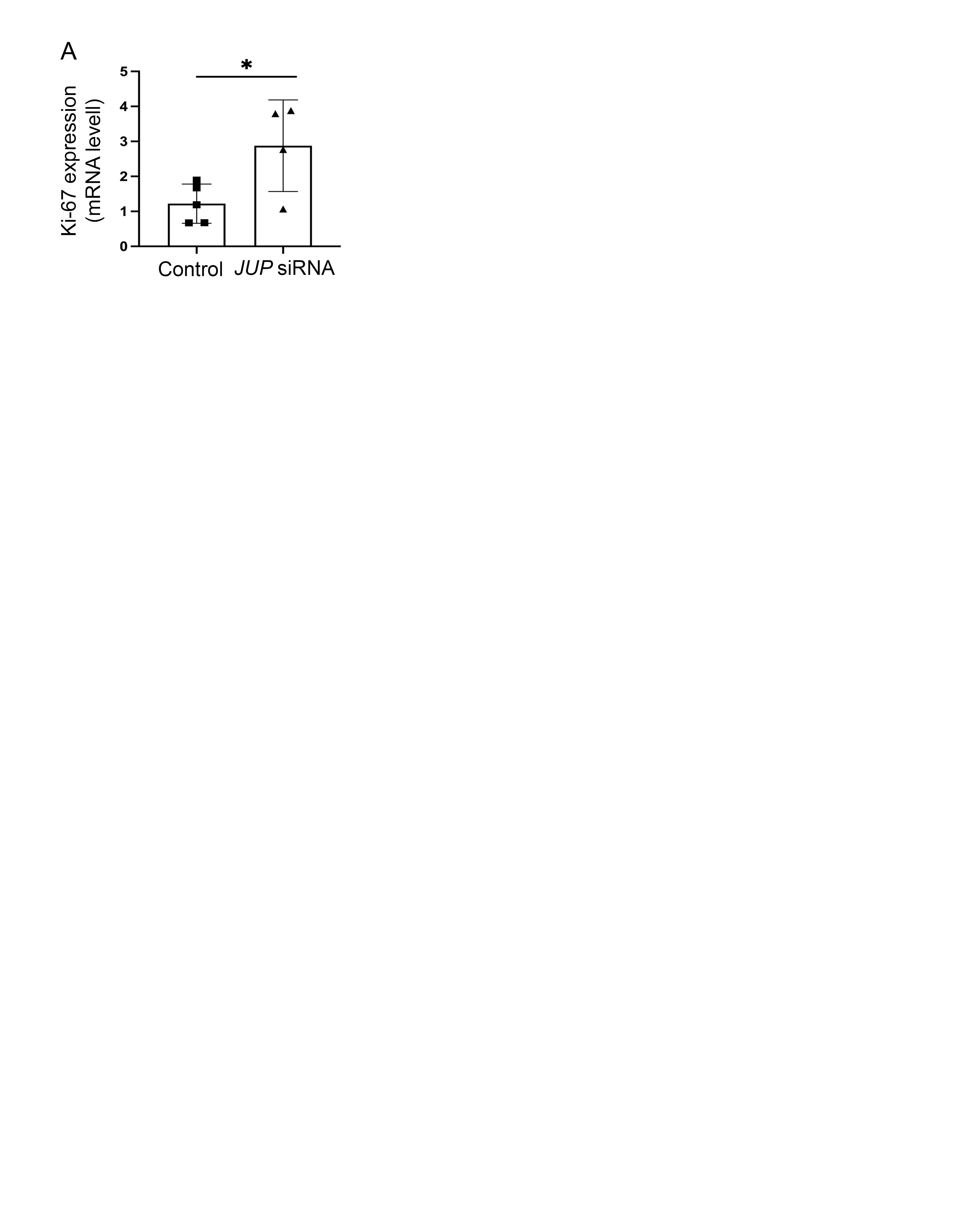

Supplement: Supplementary file 1 — Supplementary Material 1 [file 12032_2026_3277_MOESM1_ESM.jpg]
